# Supplementary material for: Structural studies reveal flexible roof of active site responsible for ω-transaminase CrmG overcoming by-product inhibition
Source: Commun Biol. 2020 Aug 19;3:455. doi: 10.1038/s42003-020-01184-w (PMC7438487; doi:10.1038/s42003-020-01184-w)
Supplement: Supplementary file 1 — Supplementary Information [file 42003_2020_1184_MOESM1_ESM.pdf]

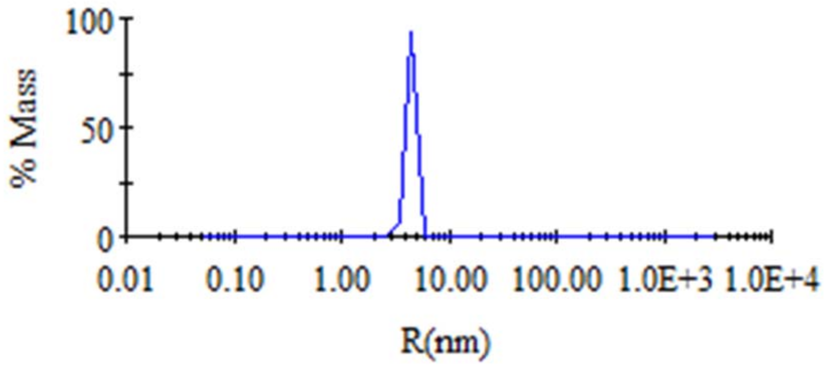

R(nm)	%Pd	MW (kDa)
**4.5**	**6.3**	**114**

Supplementary **Figure1.** Analysis of solution property of CrmG using DLS.

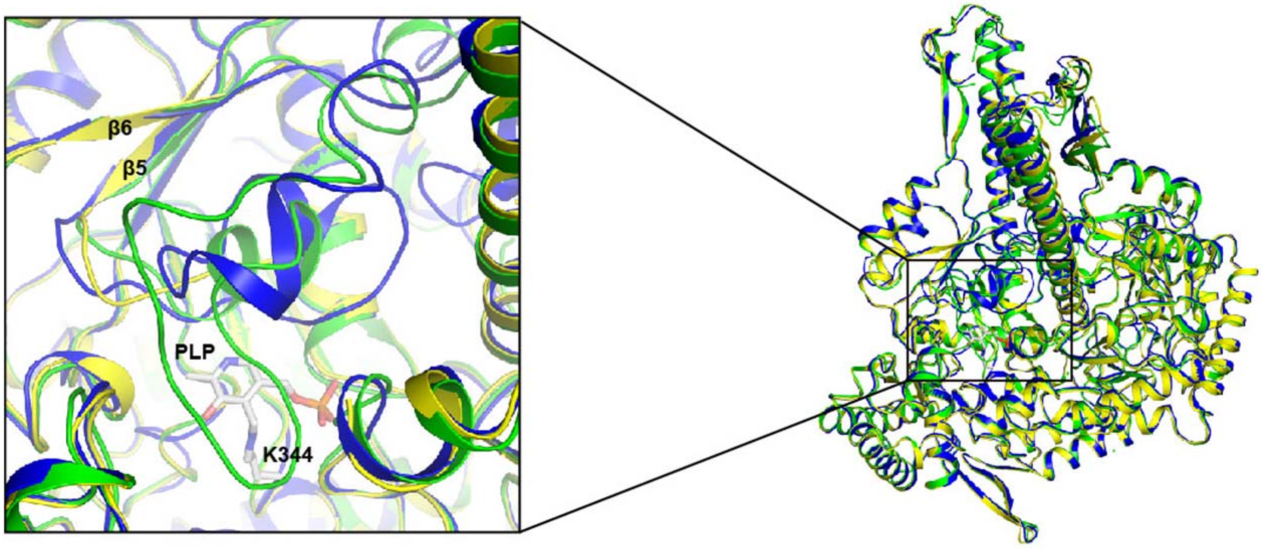

Supplementary **Figure 2.** Structures of apo CrmG are superposed on PLP-bound CrmG (PDB code 5DDS). Structures of apo CrmG in C2 space group and I222 space group are colored in yellow and green, respectively; structure of PLP-bound CrmG is colored in blue, internal aldimine is shown as white stick.

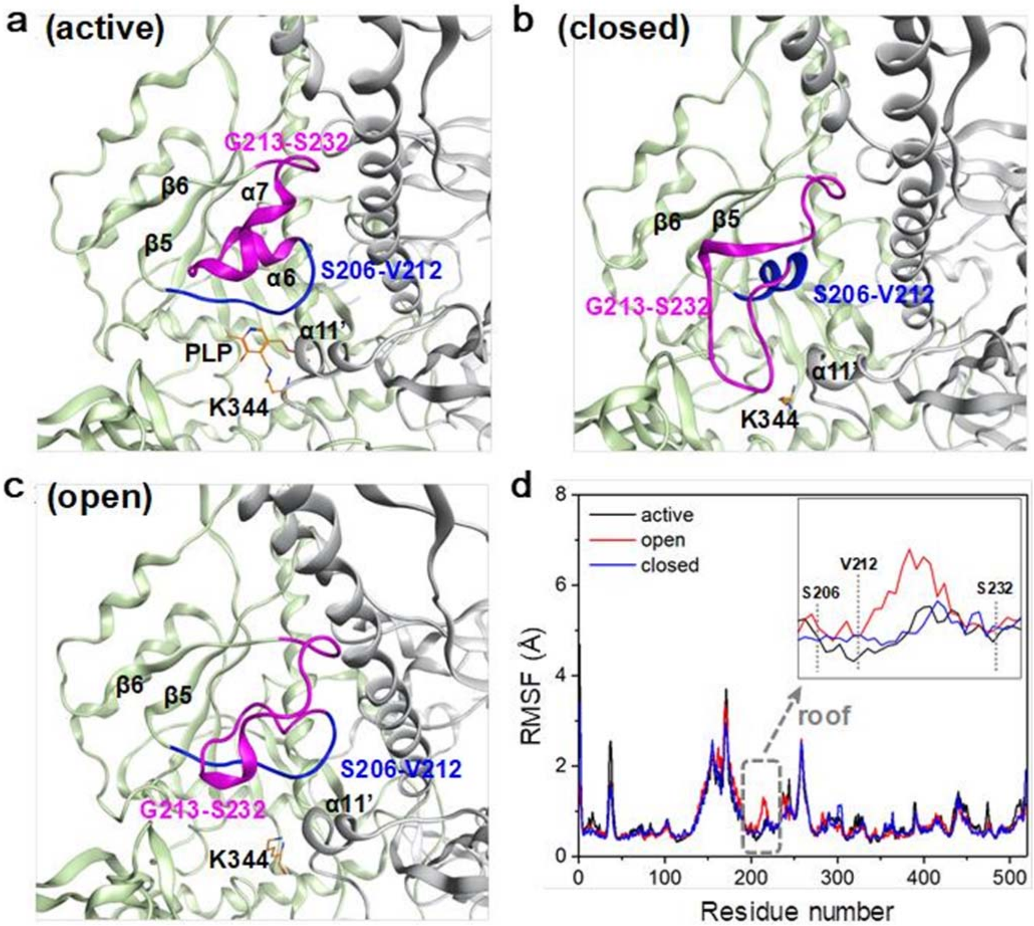

Supplementary **Figure 3.** The ensemble structures for the "roof" region in CrmG from MD simulations in active form (a), closed apo form (b) and open apo form (c). The two monomers are color by light green and gray, segments of S206-V212 and G213-S232 are color in blue and magenta, K344 and PLP are shown as orange stick. (d) RMSF of CrmG in active, open and closed form. The "roof" region is shown in expanded view.

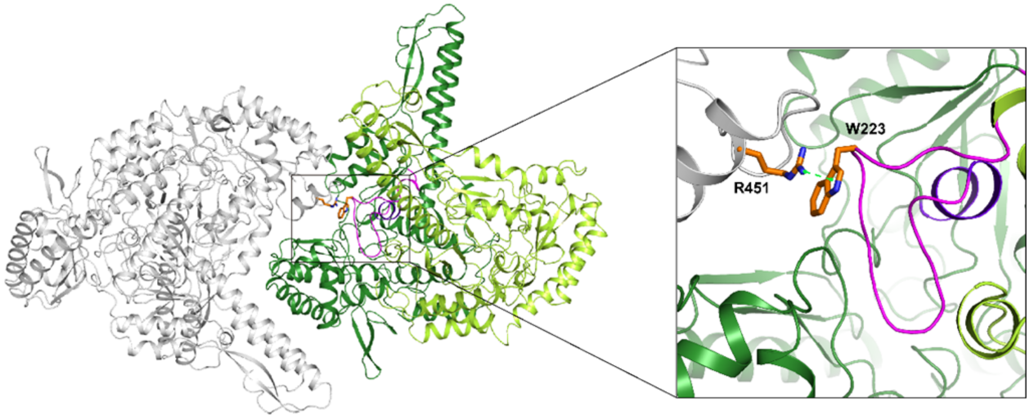

Supplementary **Figure 4.** Residue W223 from the closed pocket forms a cation-π interaction with R451 from a symmetry-related molecule in apo CrmG with space group I222. The symmetry-related molecule is shown as white cartoon.

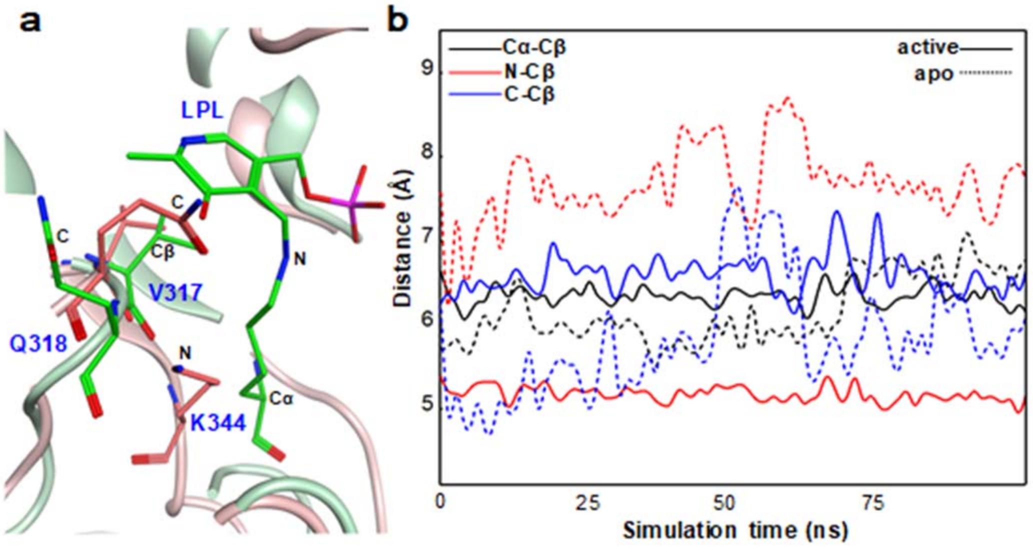

Supplementary **Figure 5.** (a) Comparison of β10/α10 loop and K344 on apo CrmG (pink) with that on PLP-Bound CrmG (light green) from MD simulations, and key distances tracking along the MD trajectory. V317, Q318, K344, and PLP are shown as stick, some key atoms that were used to track the segment movement are also labeled. (b) Distance of Cα-Cβ (black line) and N-Cβ (red line) represent the main chain and side chain shift of K344, and C-Cβ (blue line) represents the positional shift of Q318. The solid lines represent the distance changes in active form of CrmG and dashed lines represent those in apo form.

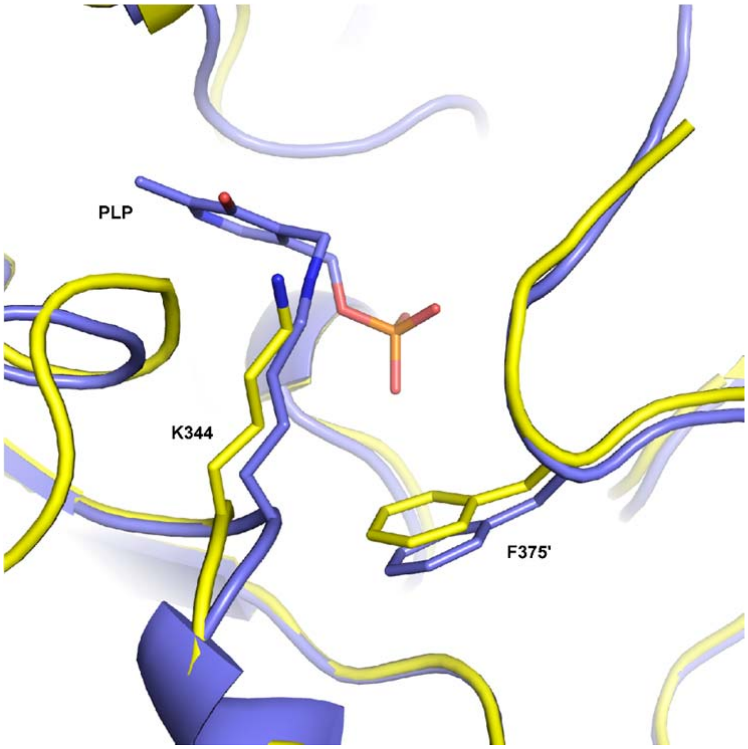

Supplementary **Figure 6.** Catalytic lysine K344 moves away from F375′ in apo CrmG (yellow) compared with that in PLP-bound CrmG (Blue).

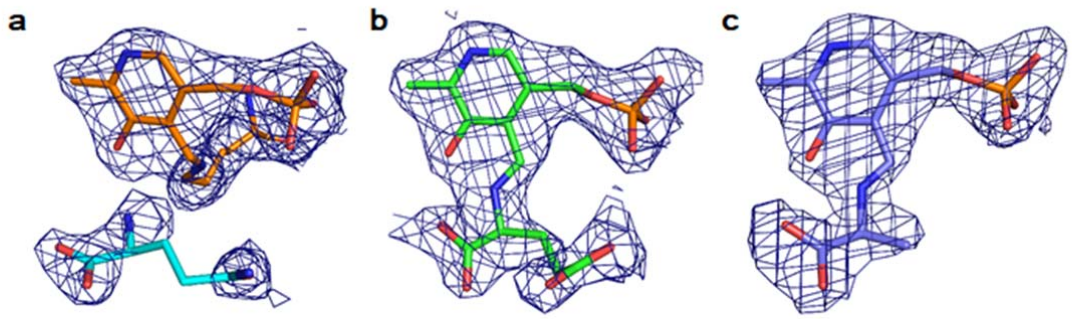

Supplementary **Figure 7.** Electron density map for L-Gln and PLP (a), PLP-Glu (b), and PLP-Ala (c). The 2Fo−Fc electron density map contoured at 1.0 σ is shown as blue mesh.

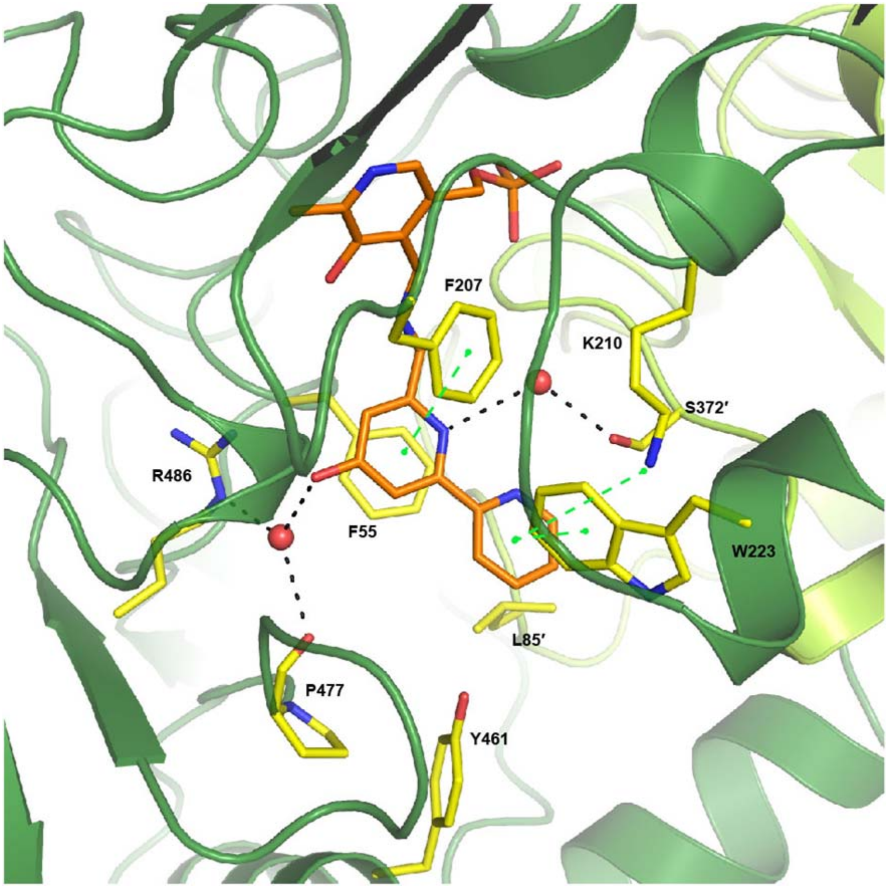

Supplementary **Figure 8.** Detailed interactions of CRM M with CrmG. PMP-CRM M is shown as orange stick; critical residues for CRM M binding are shown as yellow sticks; water molecules involving in CRM M binding are shown as red spheres. Cation-π interaction is shown as green dash, and hydrogen bonds are shown as black dash.

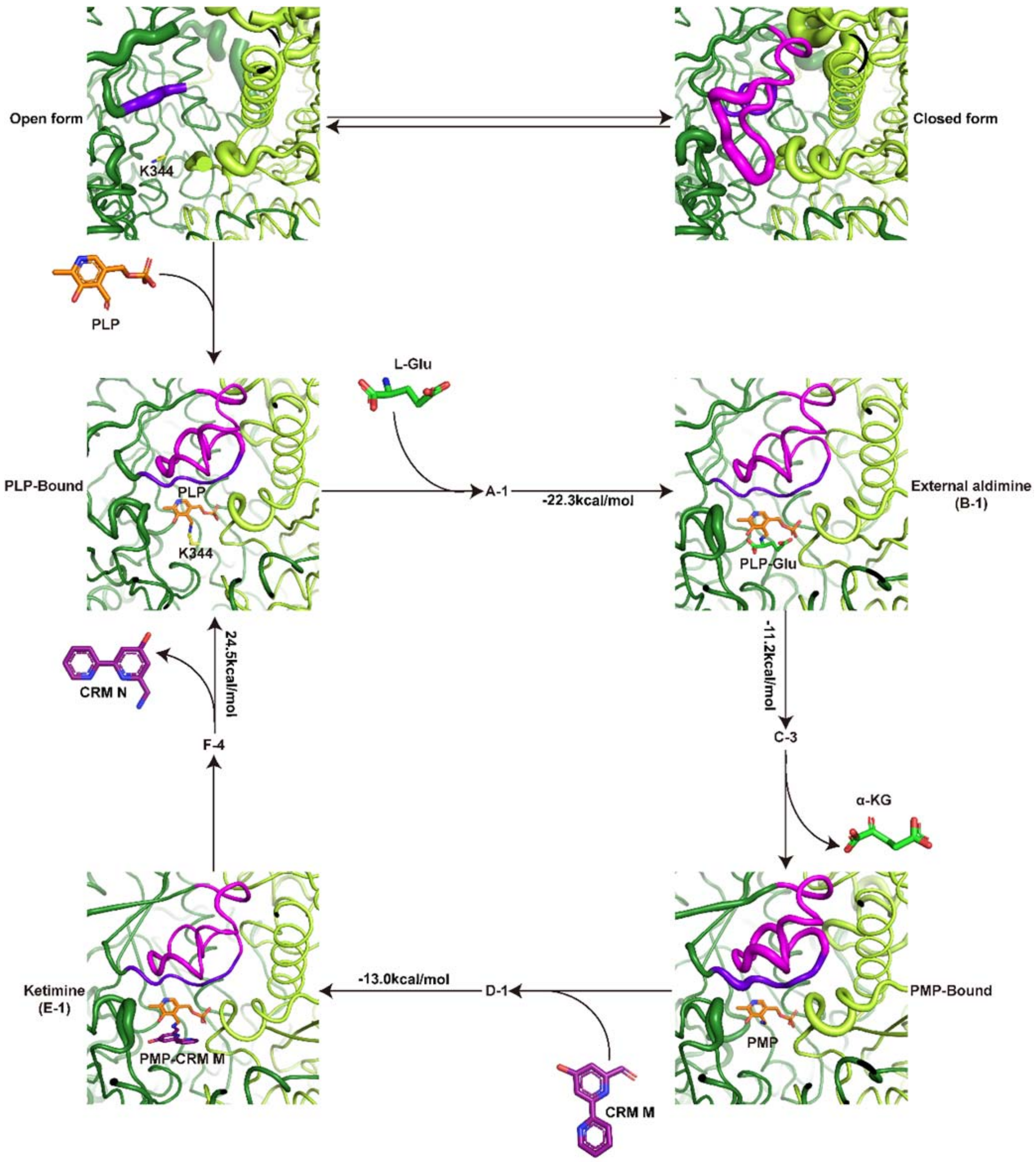

Supplementary **Figure 9.** Proposed mechanism of CrmG eliminating by-product inhibition. In apo CrmG, the active site exhibits two states: open form and closed form. In open form, PLP can enter into the active site and induce the activation of CrmG. In PLP-bound CrmG, amino donor enters into the active site, and then first half reaction occurs. The amino group of amino donor is transferred to PLP, generating PMP and by-product. The by-product is then released from the active site. PLP converting to PMP results in the increase of the B factor of the roof region (colored purple blue and magenta) of the active site. The flexibility of the active site will reduce by-product inhibition. However, amino acceptor CRM M can form strong interactions with and stabilize the roof region of active site, which facilitate CRM M to accept

amino group from PMP. The dimeric CrmG structures were presented as B factor putty, the cartoon thickness represents the B-factors within molecule, monomer 1 is shown in deep green and monomer 2 in light green, the roof of active is colored purple blue and magenta. Stages A-1, B-1, C-3, D-1, E-1 and F-4 correspond to those in Figure 6A.

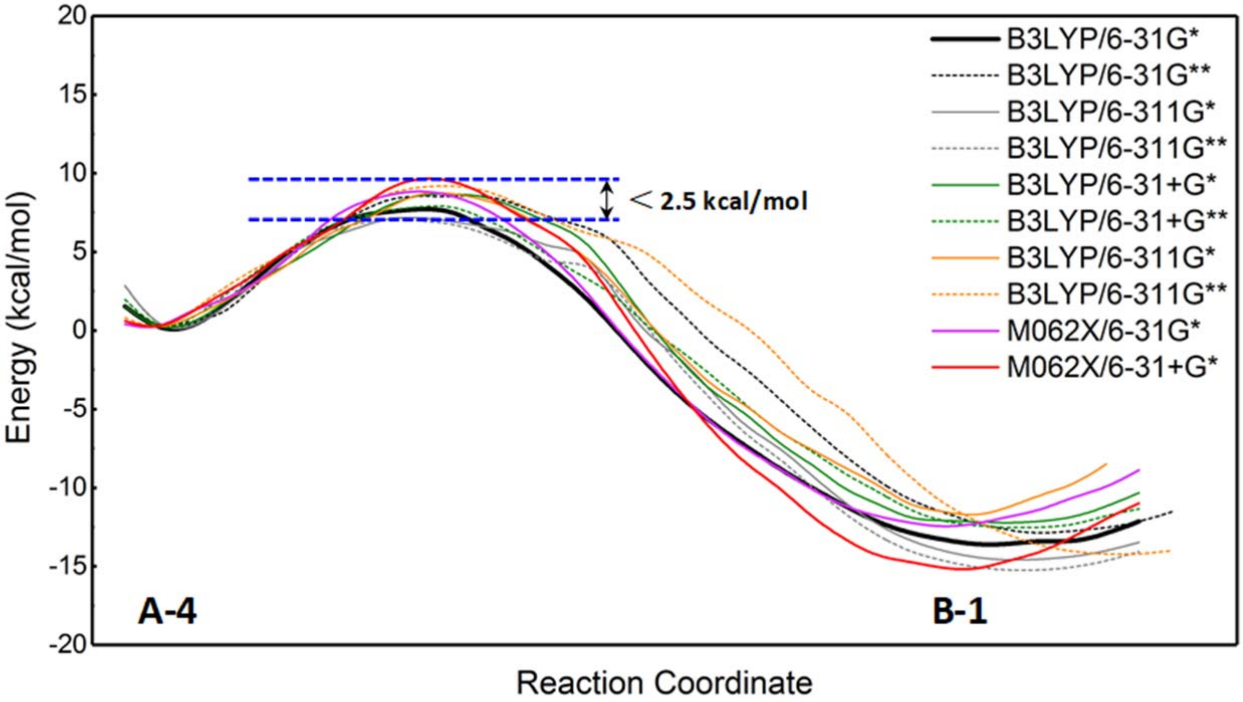

Supplementary **Figure 10.** Benchmark tests on the computational level (functionals and basis sets) for a randomly selected reaction step (A4-B1). Results confirm the reliability of the B3LYP/6-31G* level in our current QM/MM simulations on CrmG.

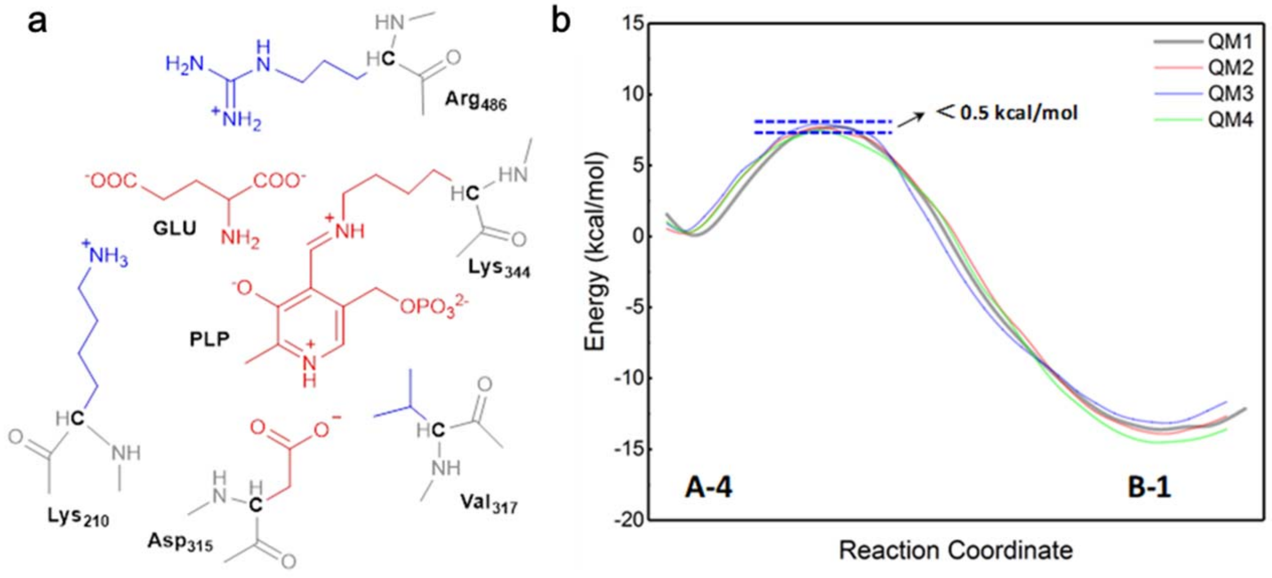

Supplementary **Figure 11.** Benchmark tests on the choice of QM region. The reaction step of A4-B1 is selected. (a) Partition of QM region, QM1 contains the substrate Glu, cofactor PLP, Asp315 and Lys344. QM2 contains QM1 and Lys210, QM3 contains QM1 and Val317, QM4 contains QM1 and Arg486. Atoms colored in red and blue are selected as QM atoms in our current QM/MM simulation, the carbon atoms in bold are the pseudoatoms that used to link the QM and MM region. (b) Energy surfaces of the selected reaction step calculated with different QM regions. Results confirm the reliability of QM1 as the QM region in our current QM/MM simulations on CrmG.

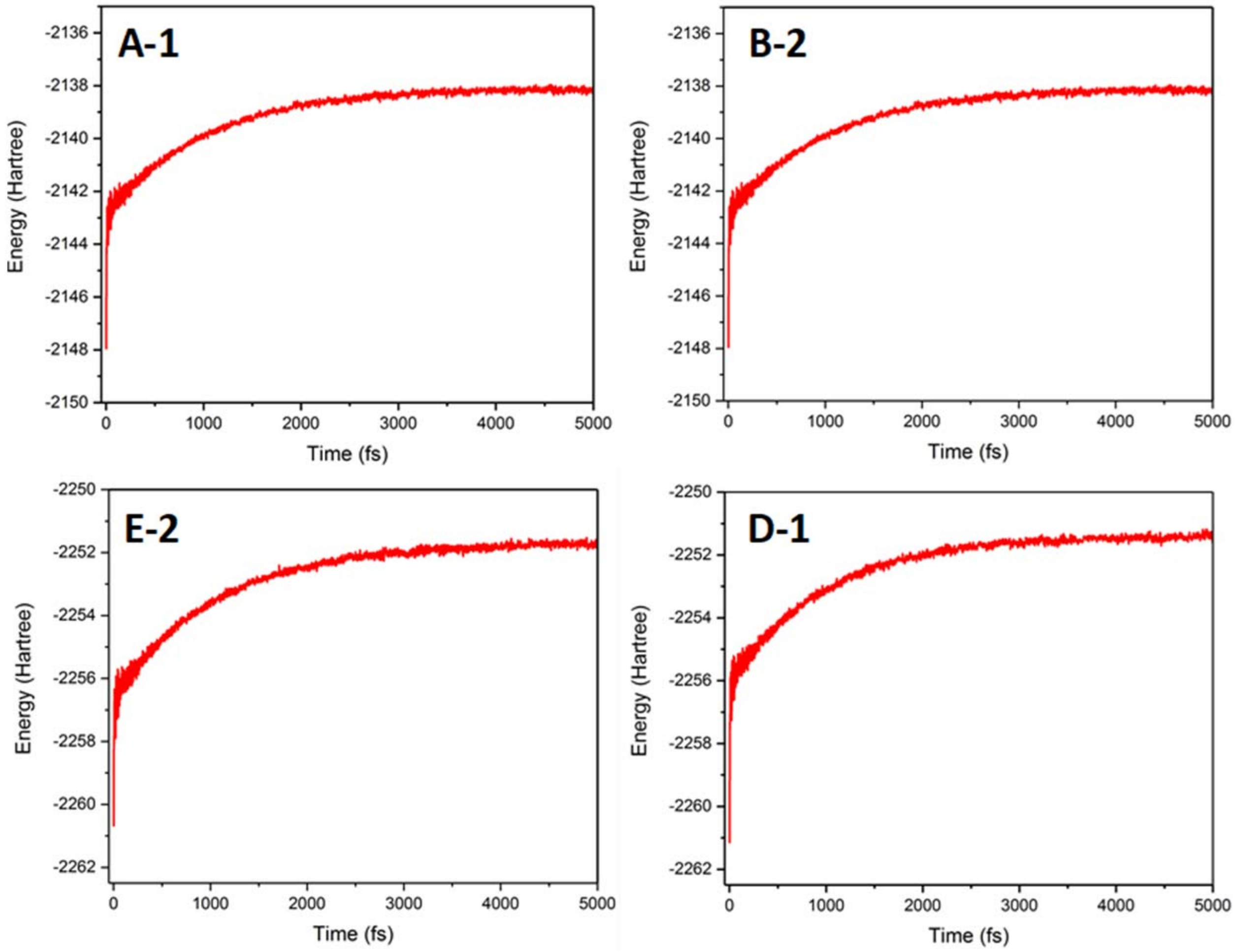

Supplementary **Figure 12.** Benchmark tests on the equilibration time scale of the QM/MM MD simulations. All the four tested systems (A-1, B-2, E-2 and D-1) are equilibrated after 2 ps during the 5 ps QM/MM MD simulations, which confirms the reliability of the equilibration time scale in our current QM/MM simulations on CrmG.

Supplementary Table 1. Convention of CRM M into CRM N

Amino donor	Conversion (%)	
	4 min	1h
L-Glu	21.4	>99
L-Gln	15.3	>99
L-Ala	8.0	>99

The enzymatic assays were performed in 50 mM Tris-HCl buffer for an hour by incubating 200 μM CRM M with 4 μM CrmG and 2 mM PLP, in the presence of 10 mM of amino donor. HPLC was used to analyze the enzymatic activity, as previously described [1].

Supplementary References

1. Zhu, Y. et al. Biochemical and Structural Insights into the Aminotransferase CrmG in Caerulomycin Biosynthesis. *ACS Chem Biol* **11**, 943-52 (2016).
