# Supplementary material for: Structural studies reveal flexible roof of active site responsible for ω-transaminase CrmG overcoming by-product inhibition
Source: Commun Biol. 2020 Aug 19;3:455. doi: 10.1038/s42003-020-01184-w (PMC7438487; doi:10.1038/s42003-020-01184-w)
Supplement: Supplementary file 2 — Description of Additional Supplementary Files [file 42003_2020_1184_MOESM2_ESM.pdf]

Description of Additional Supplementary Files

File Name: **Supplementary Data 1**
Description the source data for Fig. 1b-d, Fig. 4a-b, and Fig. 5d
